# De Garengeot's Hernia: Two Case Reports with Correct Preoperative Identification of the Vermiform Appendix in the Hernia

**DOI:** 10.1155/2016/2424657

**Published:** 2016-12-14

**Authors:** Zhaosheng Jin, Muhammad Rafiz Imtiaz, Henry Nnajiuba, Suzette Samlalsingh, Akinyede Ojo

**Affiliations:** Department of Surgery, King George Hospital, Barking, Havering and Redbridge NHS Trust, Romford, UK

## Abstract

We present two cases of incarcerated de Garengeot's hernia. This anatomical phenomenon is thought to occur in as few as 0.5% of femoral hernia cases and is a rare cause of acute appendicitis. Risk factors include a long pelvic appendix, abnormal embryological bowel rotation, and a large mobile caecum. In earlier reports operative treatment invariably involves simultaneous appendicectomy and femoral hernia repair. Both patients were correctly diagnosed preoperatively with computed tomography (CT). Both had open femoral hernia repair, one with appendectomy and one with the appendix left in situ. Both patients recovered without complications. Routine diagnostic imaging modalities such as ultrasonography and standard CT have previously shown little success in identifying de Garengeot's hernia preoperatively. We believe this to be the first documented case of CT with concurrent oral and intravenous contrast being used to confidently and correctly diagnose de Garengeot's hernia prior to surgery. We hope that this case report adds to the growing literature on this condition, which will ultimately allow for more detailed case-control studies and systematic reviews in order to establish gold-standard diagnostic studies and optimal surgical management in future.

## 1. Introduction

Femoral hernias containing the appendix were named de Garengeot's hernia by Akopian and Alexander in 2005 after the Parisian surgeon Rene Jacques Croissant de Garengeot, who was credited for reporting the case of femoral hernia containing the appendix [1]. This phenomenon is thought to occur in 0.5% to 3% of femoral hernia cases, with a female : male ratio of around 6 : 1 [2, 3].

Here we present two cases of de Garengeot's hernia managed and followed up in our unit. In both cases preoperative CT was utilised to reach the correct diagnosis. Subsequently both cases were successfully operated on and recovered postoperatively with no complications.

## 2. Case 1

58-year-old woman was referred by her General Practitioner to the Emergency Department with a one-week history of a moderately painful irreducible lump in her right groin, associated with nausea and vomiting. She did not have any urinary or bowel symptoms. She last opened her bowel earlier that day. She was known previously to have a spontaneously reducible lump in the right groin suggestive of a hernia.

The patient's medical history included hypercholesterolaemia treated with statins, menopausal symptoms treated with hormone replacement therapy, and pending investigation of a liver lesion. She had no known allergies.

On assessment the patient was in painful distress, she was tachycardic but otherwise her vital signs were stable. Her abdomen was soft and nontender; there was a palpable, nonerythematous lump in the right groin which was tender to touch and irreducible. The patient had marginally raised amylase of 57 iu/L and C-reactive protein (CRP) of 19 mg/L; her full blood count and renal and liver function tests were within normal limits.

The patient had an abdominal radiograph which was unremarkable. A groin ultrasound scan showed an approximately 5.2 × 2.7 cm cystic abnormality with a small communication with the abdominal cavity, suggestive of a femoral hernia.

**Figure 1:**
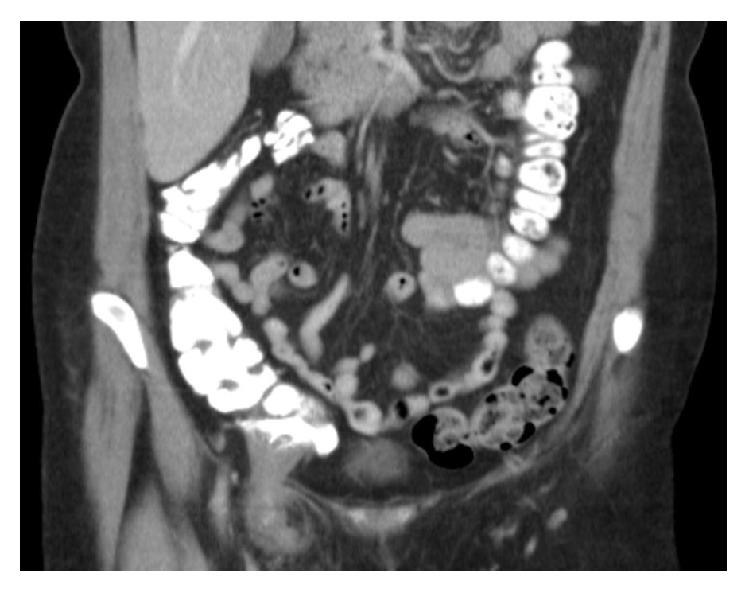
Preoperative abdominal CT image of the patient. Note the oral contrast in the colon, past the ileocaecal junction.

Patient then had an abdominal CT scan with oral and intravenous contrast (Figure 1). This was reported as a right sided femoral hernia and the caecum and the ileocaecal junction were in close proximity to the hernial orifice. A tubular structure was seen in the hernial sac which did not take up the oral contrast; this was reported as an appendix in a femoral hernia.

The patient was taken to the operating theatre for an emergency right sided exploration and hernia repair under general anaesthesia. An infrainguinal transverse incision was made over the lump and the hernial sac was dissected free. The sac was opened revealing a congested appendix and caecum. The perfusion however normalised when the neck of the hernia was released. There was no evidence of perforation of the caecum or the appendix neither was there a periappendiceal collection. An appendicectomy was then done and the base was buried using purse-string technique with 3-0 absorbable polydioxanone suture. The reduction of the caecum back into the abdominal cavity proved challenging due to the narrowness of the femoral hernia defect which was therefore dilated, enabling the caecum to be manually reduced. The decision was made to repair the hernia defect with interrupted 3-0 polypropylene suture instead of mesh as a resection had been undertaken. The skin was closed with 3-0 reabsorbable poliglecaprone 25 suture and a pressure dressing applied. The postoperative recovery was uneventful.

The patient went home on day one after the operation. The resected specimen was sent off for histological analysis, which did not show any evidence of appendicitis. At six-month follow-up patient did not have any postoperative complications.

## 3. Case 2

A 78-year-old female was referred as an emergency by her General Practitioner with a two-week history of a painless irreducible lump in the right groin. She had a long history of constipation but no recent change in her bowel habit, nausea, vomiting, or pain.

She has a medical history of asthma, hypertension, hypercholesterolaemia, Type 2 diabetes, arthritis, and deep vein thrombosis for which she was on enoxaparin.

On assessment the patient appeared well, her vital signs were stable, and her abdomen was soft and nontender. The lump was in her right groin and was tender and irreducible. The patient's inflammatory markers and renal and liver function tests were within normal limits.

An ultrasound scan showed a cystic abnormality measuring 4 × 2.2 cm in the right groin, with communication to the abdomen suggestive of an irreducible hernia with fluid and bowel loop within the hernial sac.

Abdominal CT reported a right groin hernia measuring 5.0 × 3.0 cm, containing fluid, fat, and a vermiform appendix. There was an incidental finding of chronic sigmoid diverticular disease.

The patient was taken to theatre for a right sided exploration and hernia repair under general anaesthesia. A transverse incision was made over the lump, which revealed an incarcerated femoral hernia. The sac contained a healthy appendix which was easily reducible. As the appendix was not inflamed, it was decided not to perform an appendectomy. The femoral hernia defect was repaired using interrupted nylon suture, the skin was closed with polyglactin 910 suture, and a pressure dressing was applied to minimise seroma formation and maintain haemostasis.

The patient made an uneventful recovery and went home after two days. At a three-month follow-up she did not report any postoperative complications.

## 4. Discussion

The vermiform appendix is a blind ended tubular structure connected to the caecum. In embryological development, the caecum-appendix complex develops at 6–10 weeks from an outgrowth on the midgut named the “bud of the caecum.” Because of the relatively fast growing speed of the midgut, it undergoes regular rotation during embryonic development, and as it elongates the caecum-appendix complex migrates caudally [4]. Depending on the movement of the caecum, the appendix can assume various end-positions, most common of which are pelvic and postileal ones [5]. The varying degrees of rotation of the midgut during development, the size and position of the caecum, and having a pelvic appendix are all proposed risk factors for developing de Garengeot's hernia [6, 7].

Femoral hernia cases occur through the femoral ring, into the femoral canal. They are an uncommon cause of groin lumps which account for 3–5% of abdominal hernias, having a male : female ratio of 1.8 : 1 [8]. The femoral canal is a compartment of the femoral sheath which is bordered anterosuperiorly by the inguinal ligament, medially by the lacuna ligament, laterally by the femoral vein, and posteriorly by the pectineal ligament. Due to the small potential space in the femoral canal, femoral hernias are much more likely to become incarcerated and strangulated. In the case of de Garengeot's hernia, this can result in appendicitis and eventually perforation and abscess formation. Due to the vestigial nature of the appendix, strangulation does not result in mechanical obstruction, although ileus can occasionally develop secondary to inflammation. The tight neck of the femoral canal acts as a seal which limits inflammation and infection in the hernia sac; therefore perforation of the appendix in the hernia sac will rarely present with peritonitis. It has even been reported that, after the spontaneous reduction of a perforated appendix, the hernia neck seals off the infected collection, preventing peritoneal involvement [9].

The presentations of both of our cases are that of a typical groin hernia, with irreducible, tender lumps in the groin. Both patients were systemically well with no bowel symptoms; this is despite patient 1 having an obstructed and partially strangulated hernia. The most common presentation of de Garengeot's hernia from previous reports is that of an enlarging, painful, or irreducible preexisting right groin lump [10]. Occasionally patient may have generalised abdominal symptoms [3, 11] and systemic symptoms [6]. Three reported cases were associated with obstruction, one due to involvement of the small bowel [12] and two due to peritonitis and sepsis [6, 11, 13]. Very rarely the appendix can also herniate via the left groin [7, 14].

Preoperative diagnosis of de Garengeot's hernia is rare [3, 15]. Clinical examination is of limited value in identifying the content of the femoral hernia. In all of the reviewed case reports, including our case, ultrasound has not been reported as able to diagnose a de Garengeot's hernia [2, 15–18]. Depending on protocol and reporting expertise, CT has demonstrated some value, with only 4 reported cases where CT has given the correct diagnosis [17, 19–21]. Our cases are the first ones where oral plus intravenous contrast was used for the CT scan, which helped to demonstrate the lack of small and large bowel involvement in the hernia. This aided the identification of the appendix as hernial sac content. Magnetic resonance imaging has been demonstrated to correctly identify de Garengeot's hernia in two cases but, due to the general lack of emergency magnetic resonance imaging (MRI) capacity in UK district general hospitals such as ours, this imaging modality is of limited clinical value [3, 22].

Due to the difficulty in obtaining a preoperative diagnosis, all of the reported cases of de Garengeot's hernia have been diagnosed through surgical exploration, which introduces considerable selection bias in the review of treatment options. Similar to our cases, most cases are done through a groin incision, using one of the femoral hernia repair approaches [23, 24]. However, there have been cases where the hernial sac and the appendix are reduced into the peritoneal cavity via midline laparotomy. This has been necessary due to the difficulty in mobilising the base of the appendix through a groin incision, or simply due to clinician choice [12, 25, 26]. One report described a case where the appendix was resected from the caecum via laparoscopy, followed by inguinal incision to remove the appendix and to repair the hernia [27]. In addition, total laparoscopic repair of de Garengeot's hernia has also been performed successfully. Two such cases have been reported, both with good postoperative outcomes [18, 28]. The appendix is removed in most cases. However, as demonstrated in patient 2, appendix which is visually confirmed healthy could be reduced and left in situ without compromising the postoperative outcome, whereas resection of a healthy appendix can lead to unnecessary infection risks [29].

Most case reports recommend that hernial defects should be repaired with nonabsorbable sutures. The main reason is said to be to reduce the risk of wound site infection, quoted in one study as high as 23% [30]. It is thought that the introduction of foreign material into a potentially contaminated surgical field could further increase the risk of infection [6, 31]. However, in recent years mesh repair has been used in some cases without any reported complications. Authors in such cases reason that it is the delay in surgical intervention rather than the method of hernia repair which influences the risk of surgical site infection [18, 29, 32]. Due to the limited number of cases, it is not possible to directly appraise the use of mesh repair in de Garengeot's hernia. There are however a number of studies looking into the use of mesh in strangulated and incarcerated inguinal hernia, which may be of some reference due to the similarity in the composition of the hernia content and the surgical management involved. In a systematic review of 413 patients, Hentati concluded that there are no significant differences in the rate of surgical site infection between mesh and suture repair; this was true for both cases with and without bowel resection. On the other hand, the rate of recurrence was significantly lower in the mesh treated group [33]. Yang et al. reviewed postoperative surgical site infection rate in incarcerated inguinal hernia and found that 4 out of 103 cases of mesh repair and 5 out of 9 suture repairs developed surgical site infection [34]. Unsurprisingly, they also found that the risk of surgical site infection is significantly higher in cases involving bowel resection, supporting Nguyen's proposal that if appendectomy is not performed (in the case of healthy appendix), de Garengeot's hernias can be repaired with mesh without significantly increased risk of infection [30].

Postoperatively most patients recover without complications, with an average hospital stay of 5 days in the case reports we reviewed; this however depends on the state of the appendix during surgery, as perforated appendix is significantly more likely to lead to surgical site infection in the postoperative period.

In summary, we report two cases of de Garengeot's hernia; both were correctly diagnosed using CT with oral and intravenous contrast. In one case an open appendectomy was performed and in the other the appendix was left in situ; both had uneventful recovery. Due to the infrequency of the case, there is no standard surgical practice, and cases are managed based on first principles. The similarities in the reported cases may be of some informative value for clinicians. With increasing published case reports it may be possible to systematically review the cases and reach a consensus as to what the optimal surgical management may be.
